# 

**Published:** 1915-06

**Authors:** 


PUBLISHED MONTHLY. SUBSCRIPTION $1.00

### ATLANTA JOURNAL-RECORD OF MEDICINE

LIBRARY

Successor to { Atlanta Medical and Surgical Journal, Established 1855, and Southern Medical Record, Established 1870. } 62nd Year

Vol. LXII ATLANTA, GA., JUNE, 1915. No. 3

